# Salvage pancreaticoduodenectomy for localized recurrence after resection of ascending colon cancer: a case report

**DOI:** 10.1007/s12328-026-02420-2

**Published:** 2026-08-14

**Authors:** Rihito Nagata, Nobuyuki Takemura, Masahiko Komagome, Michio Kogure, Ryosuke Satake, Natsuko Takayanagi, Yasuhiro Nunokawa, Akira Maki, Yoshifumi Beck

**Affiliations:** 1 Hepatobiliary and Pediatric Surgery Division, Department of Surgery, Saitama Medical Center, Saitama Medical University, 1981 Kamoda, Kawagoe, Saitama, 350-8550 Japan; 2 Department of Surgery, Ikebukuro Hospital, Seikokai Medical Corporation, 3724-6 Kasahata, Kawagoe, Saitama, 350-1175 Japan; 3 Department of Pathology, Saitama Medical Center, Saitama Medical University, 1981 Kamoda, Kawagoe, Saitama, 350-8550 Japan

**Keywords:** Colorectal cancer, Local recurrence, Pancreaticoduodenectomy

## Abstract

A 66-year-old man underwent right hemicolectomy for ascending colon cancer with direct invasion of gallbladder. Numerous aberrant proliferative vessels from the duodenum were noted. The pathological diagnosis was T4bN0M0, Stage IIC, with a KRAS G12D mutation. He received four cycles of adjuvant chemotherapy. Four months later, a mass approximately 3 cm in diameter involving the duodenum and ileum was identified and diagnosed as localized recurrence with no evidence of distant metastasis. After administration of two cycles of chemotherapy, contrast-enhanced computed tomography demonstrated tumor shrinkage. A pancreaticoduodenectomy was performed, including resection of the involved ileum, and the short segment of transverse colon. Pathological examination revealed adenocarcinoma proliferation extending across the adhesion plane between the duodenum and ileum, infiltrating the muscularis propria of both intestinal walls without intraluminal exposure. These findings suggested that the tumor originated from residual cancer cells at the dissection plane of the initial surgery. No postoperative adjuvant chemotherapy was administered, in accordance with the patient’s wishes. The patient has remained disease-free for more than 7 years after the pancreaticoduodenectomy.

## Introduction

Tumor recurrence involving the resection site and surrounding peritoneum is commonly encountered after curative-intent resection for colorectal cancer. The treatment of these recurrences of colorectal cancer is primarily based on systemic therapy, and surgical resection is not currently regarded as a standard therapy in major clinical guidelines, including the National Comprehensive Cancer Network Guidelines in the United States [1] and the Japanese Guidelines for the Treatment of Colorectal Cancer [2]. However, in selected cases, surgical resection may be considered when the disease is thoroughly evaluated, and complete resection is deemed achievable with a well-planned procedure. In the present case, the patient received systemic chemotherapy prior to resection of the localized recurrence and subsequently underwent pancreaticoduodenectomy, achieving long-term disease-free survival. Although similar cases have been sporadically reported [3–5], in the present case the findings of the resected specimen suggested that the recurrent lesion represented regrowth of microscopic residual cancer cells from the initial surgery. The therapeutic response to preoperative chemotherapy demonstrated by histopathological findings, together with complete resection achieved by pancreaticoduodenectomy, may have contributed to the favorable long-term outcome.

## Case report

**Fig. 1:**
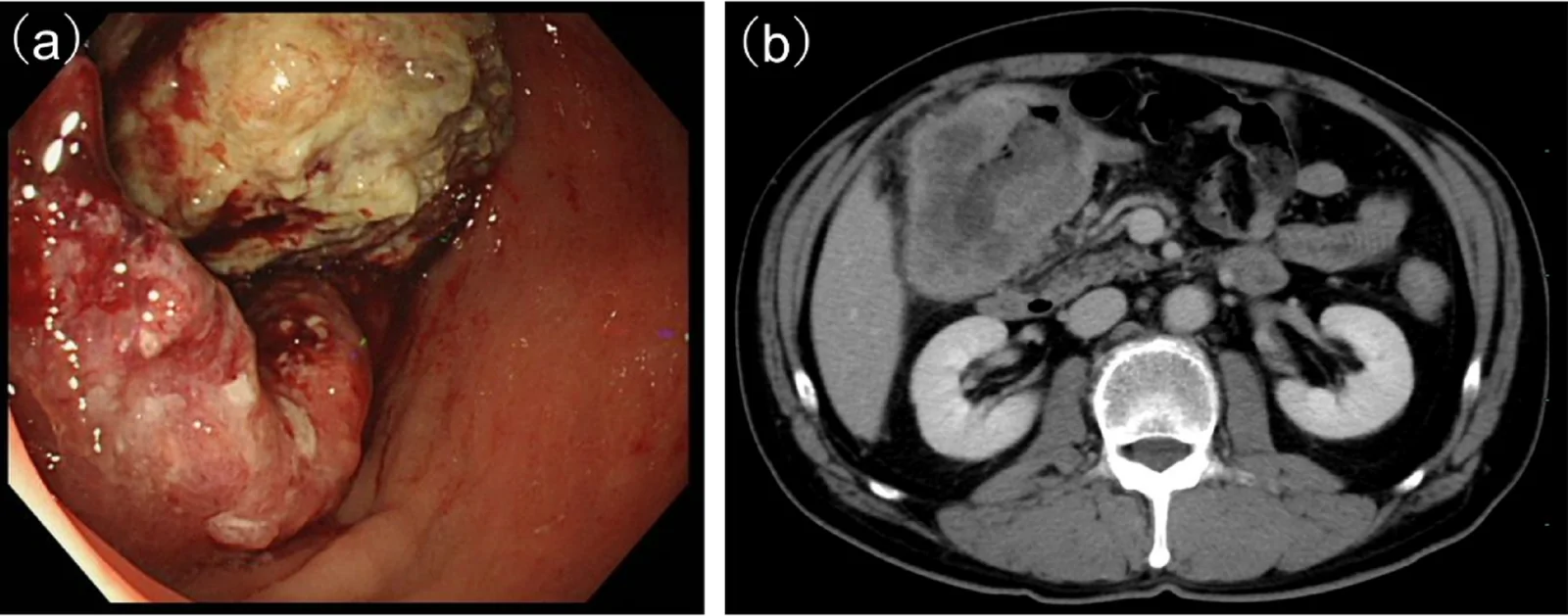
Findings of the primary tumor. **a** Colonoscopy showing a type 2 advanced tumor growing expansively and nearly filling the lumen of the ascending colon. **b** A CE-CT image marked circumferential wall thickening and enlargement of the affected bowel segment. The surrounding mesenteric fat shows stranding, suggestive of extramural invasion

A 66-year-old man presented to the referring hospital with nausea and epigastric discomfort and was diagnosed with ascending colon cancer based on colonoscopy and contrast-enhanced computed tomography (CE-CT) (Fig. 1a,b). Right hemicolectomy with cholecystectomy was performed because the gallbladder was found to be invaded by the tumor. In addition, numerous feeding vessels originating from the duodenum were observed, and part of the serosa of the duodenum was dissected during the resection.

**Fig. 2:**
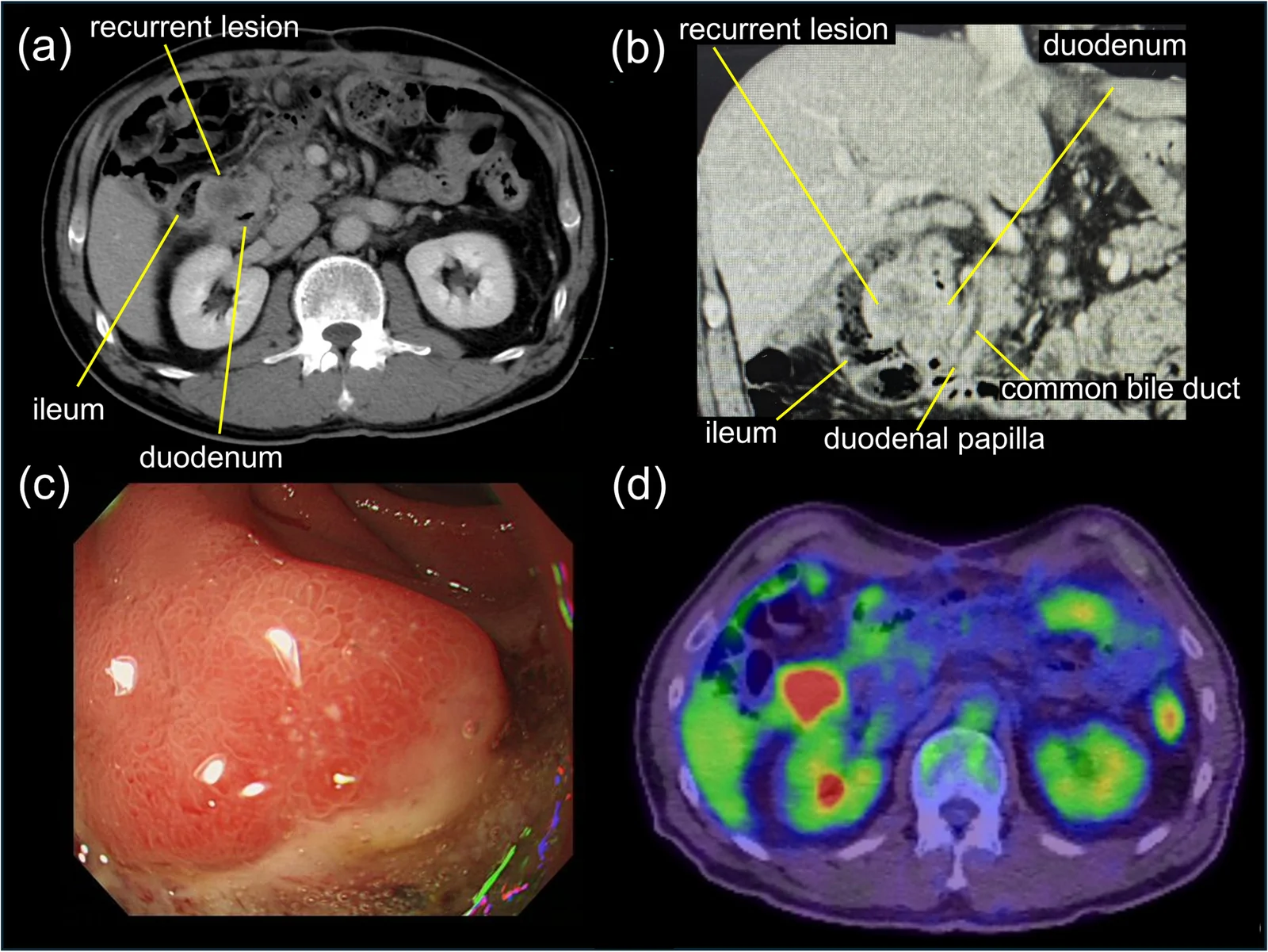
Findings of the recurrent tumor. **a** An axial CE-CT image at the time of recurrence demonstrated a low-attenuation mass located between the duodenum and the ileum, which had been repositioned to the right upper abdomen during the previous surgery. The lesion appeared to invade both adjacent ileal segments. **b** On the coronal CE-CT image, the common bile duct was mildly compressed by the tumor without evidence of obstruction, and the lesion was located in close proximity to the duodenal papilla. **c** Endoscopy revealed an elevated erythematous lesion, predominantly involving the anterior wall and extending over approximately half of the duodenal circumference. Biopsy specimens obtained from the lesion did not reveal any malignant findings. **d** ^18^F-FDG-PET showed intense uptake adjacent to the duodenum. No abnormal uptake exceeding physiological levels was observed in any other region

**Fig. 3:**
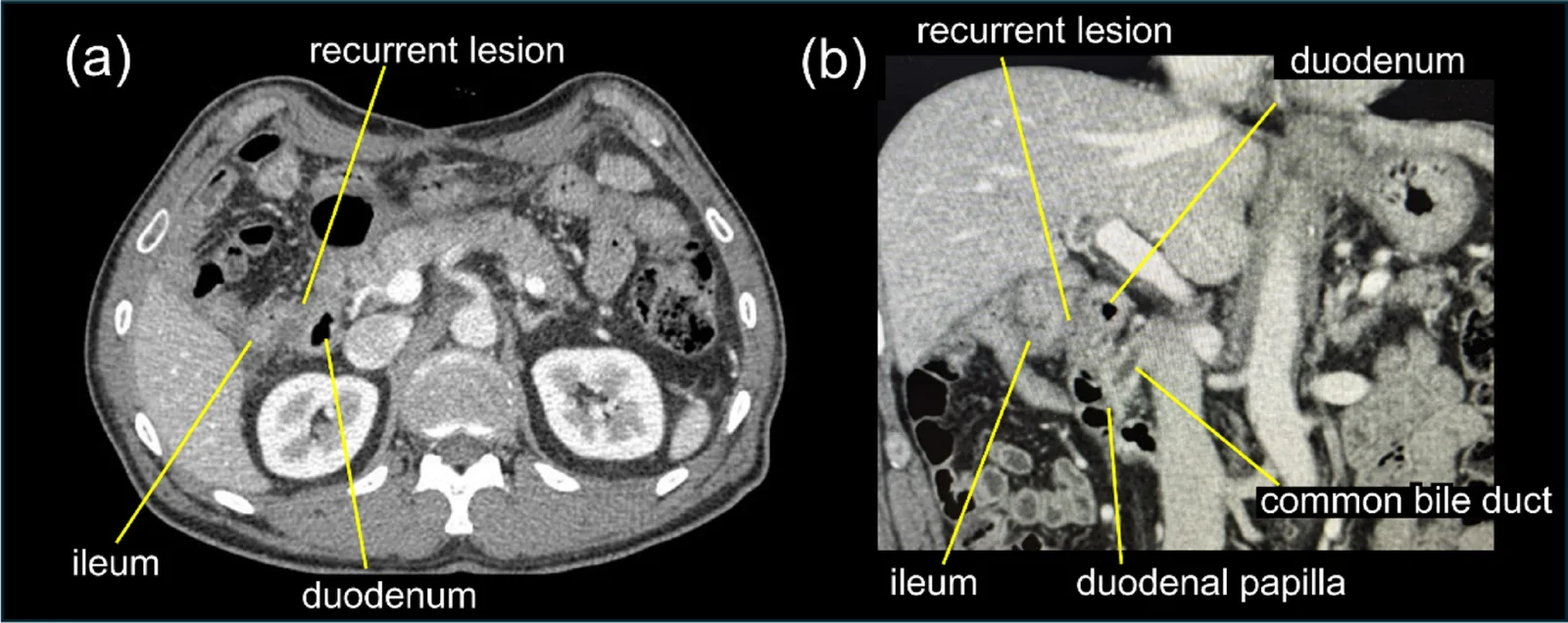
Tumor showing a partial response after chemotherapy with FOLFIRI plus bevacizumab. **a** An axial CE-CT image showed that, compared with the findings at the time of recurrence diagnosis, the tumor had decreased in size and demonstrated reduced contrast enhancement. Compression of the duodenal wall remained from the anterior to the lateral wall. **b** Coronal imaging showed improvement in the displacement of the common bile duct

**Fig. 4:**
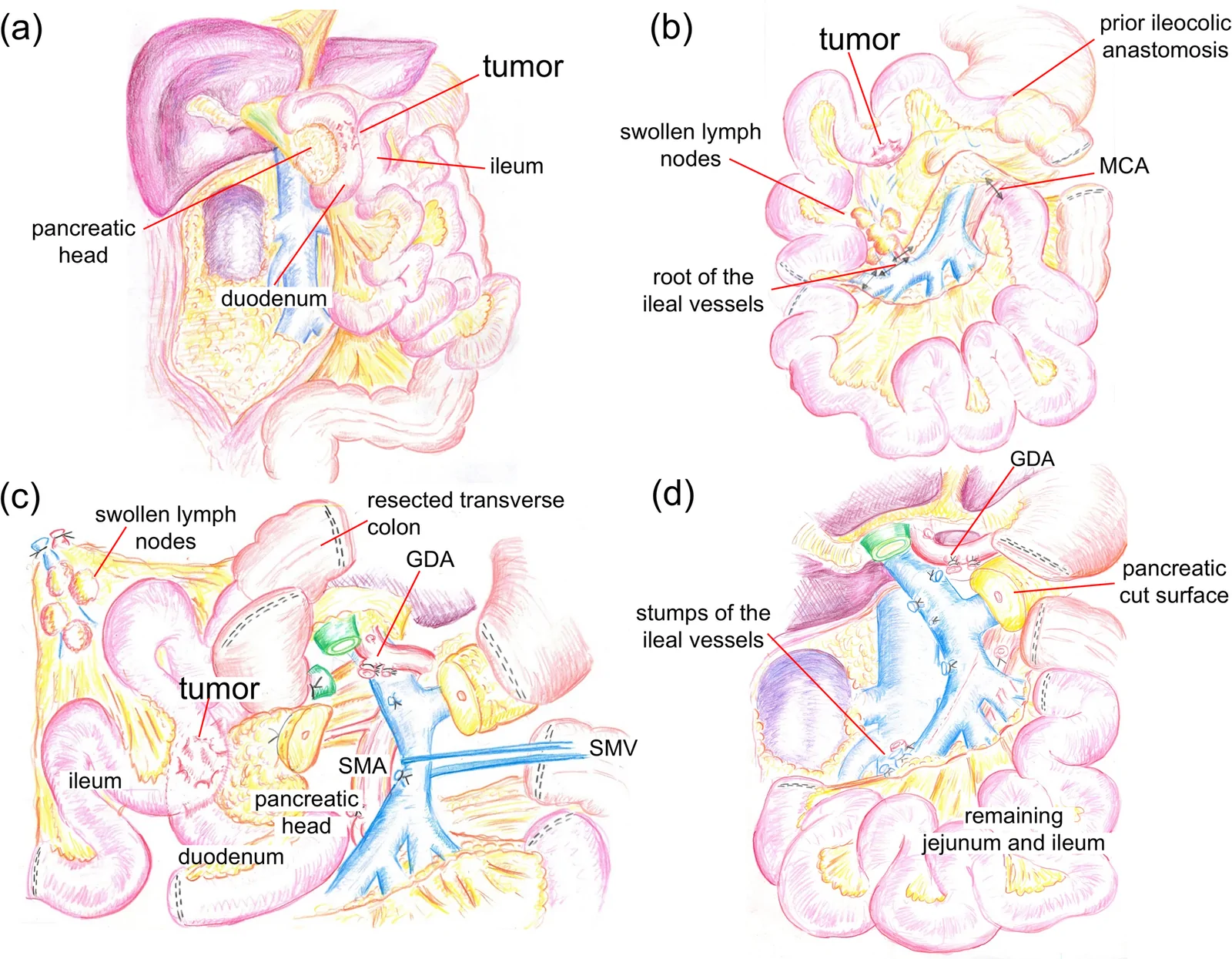
Schematic illustration of the surgical procedure. **a** Extensive mobilization of the entire small intestine and pancreatic head using the Cattell–Braasch maneuver **b** Presence of enlarged lymph nodes and determination of the extent of bowel resection. **c** Schematic illustration of pancreatic transection with en bloc resection of approximately 100 cm of the ileum and approximately 5 cm of the transverse colon. **d **Completion of pancreaticoduodenectomy, showing vascular exposure from the superior mesenteric vein to the main portal vein. GDA, gastroduodenal artery; MCA, middle colic artery; SMA, superior mesenteric artery; SMV, superior mesenteric vein

Histopathological examination revealed direct tumoral invasion into the gallbladder. The distance between the leading edge of the carcinoma and the dissected margin was approximately 3 mm. The main tumor extended close to the serosal surface of the colon, which was covered by reactive fibrin deposition; however, the tumor was not exposed, maintaining a clearance of approximately 500 μm. The pathological diagnosis was T4bN0M0, Stage IIC, according to the Union for International Cancer Control Tumor-Node-Metastasis classification. Postoperatively, four cycles of adjuvant chemotherapy consisting of capecitabine and oxaliplatin (XELOX regimen) were administered. However, 4 months later, contrast-enhanced computed tomography revealed a mass approximately 3 cm in diameter on the extraluminal side of the duodenum, suggestive of recurrence (Fig. 2a). The mass appeared to invade the adjacent ileum. Although there was no clear evidence of pancreatic parenchymal invasion, displacement of the common bile duct was observed (Fig. 2b). Endoscopy revealed an elevated erythematous lesion, predominantly involving the anterior wall and extending over approximately half of the duodenal circumference. Although no malignant cells were detected on biopsy, the lesion was clinically diagnosed as a localized recurrence (Fig. 2c). ^18^F-fluorodeoxyglucose positron emission tomography (^18^F-FDG-PET) demonstrated focal uptake at this site without evidence of distant metastasis (Fig. 2d). As systemic chemotherapy, a regimen of 5-fluorouracil, irinotecan, leucovorin, and bevacizumab (FOLFIRI + bev) was administered because the tumor harbored a KRAS G12D mutation. The patient experienced substantial adverse effects during the first course. Because surgical resection was considered at this stage, the second course was administered without bevacizumab. The tumor subsequently decreased in size and met the criteria for a partial response according to the Response Evaluation Criteria in Solid Tumors (RECIST), version 1.1 [6] (Fig. 3a,b). In addition, because the patient declined further chemotherapy, we decided to proceed with surgical resection 44 days after bevacizumab administration. At laparotomy, no gross peritoneal dissemination or ascites was observed. Peritoneal lavage cytology was not performed because extensive abdominal adhesions precluded the creation of sufficient space for lavage. After meticulous adhesiolysis, the entire small intestine and pancreatic head were mobilized in accordance with the Cattell–Braasch maneuver (Fig. 4a). Enlarged lymph nodes were identified near the middle colic artery and at the root of the ileal vessels. Frozen-section examination of the lymph node along the middle colic artery was negative; therefore, only approximately 5 cm of the transverse colon was resected to facilitate re-anastomosis. In contrast, multiple markedly enlarged lymph nodes were present at the root of the ileal vessels. Resection of this region together with the tumor-involved bowel was expected to require removal of approximately 100 cm of ileum, with at least 170 cm of small intestine expected to remain. Given the risk of a false-negative, complete tumor clearance was prioritized, and the ileum was resected en bloc without frozen-section examination. (Fig. 4b). The tumor was palpated as a firm mass on the anterior wall, slightly proximal to the duodenal papilla., and there was no intraoperative evidence sufficient to rule out invasion of the pancreatic parenchyma. In addition, a limited resection was expected to result in a large duodenal wall defect (Fig. 4c). Therefore, pancreaticoduodenectomy was completed (Fig. 4d).

**Fig. 5:**
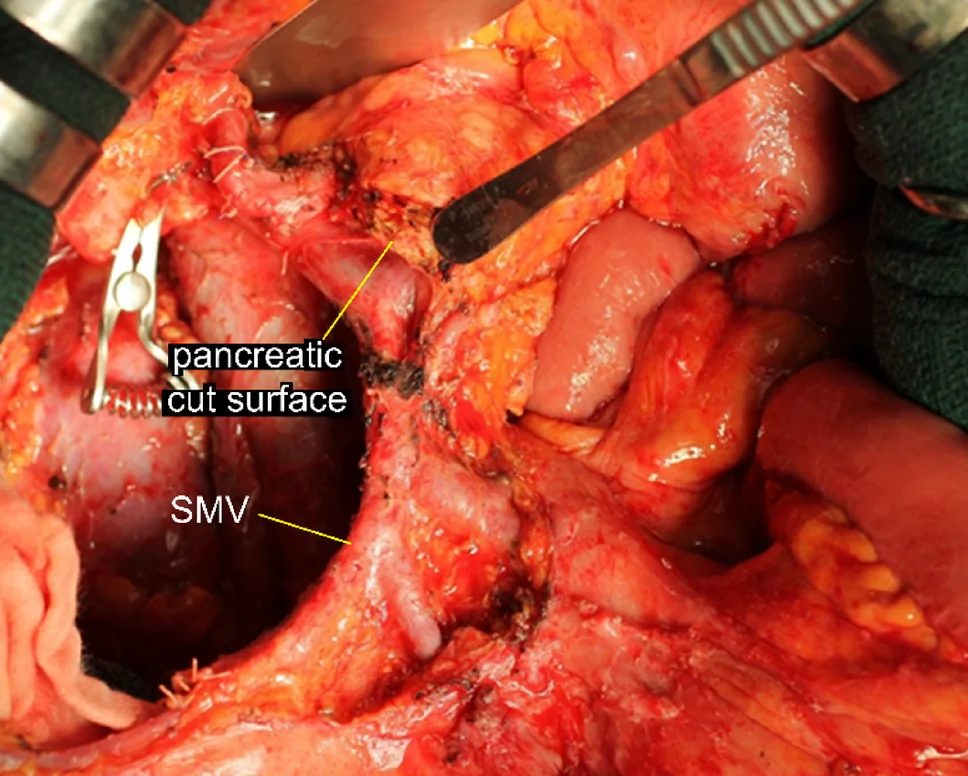
Intraoperative photograph. Image obtained after completion of specimen resection, corresponding to Fig. 4d. The transected pancreatic stump is exposed using forceps as a spatula

**Fig. 6:**
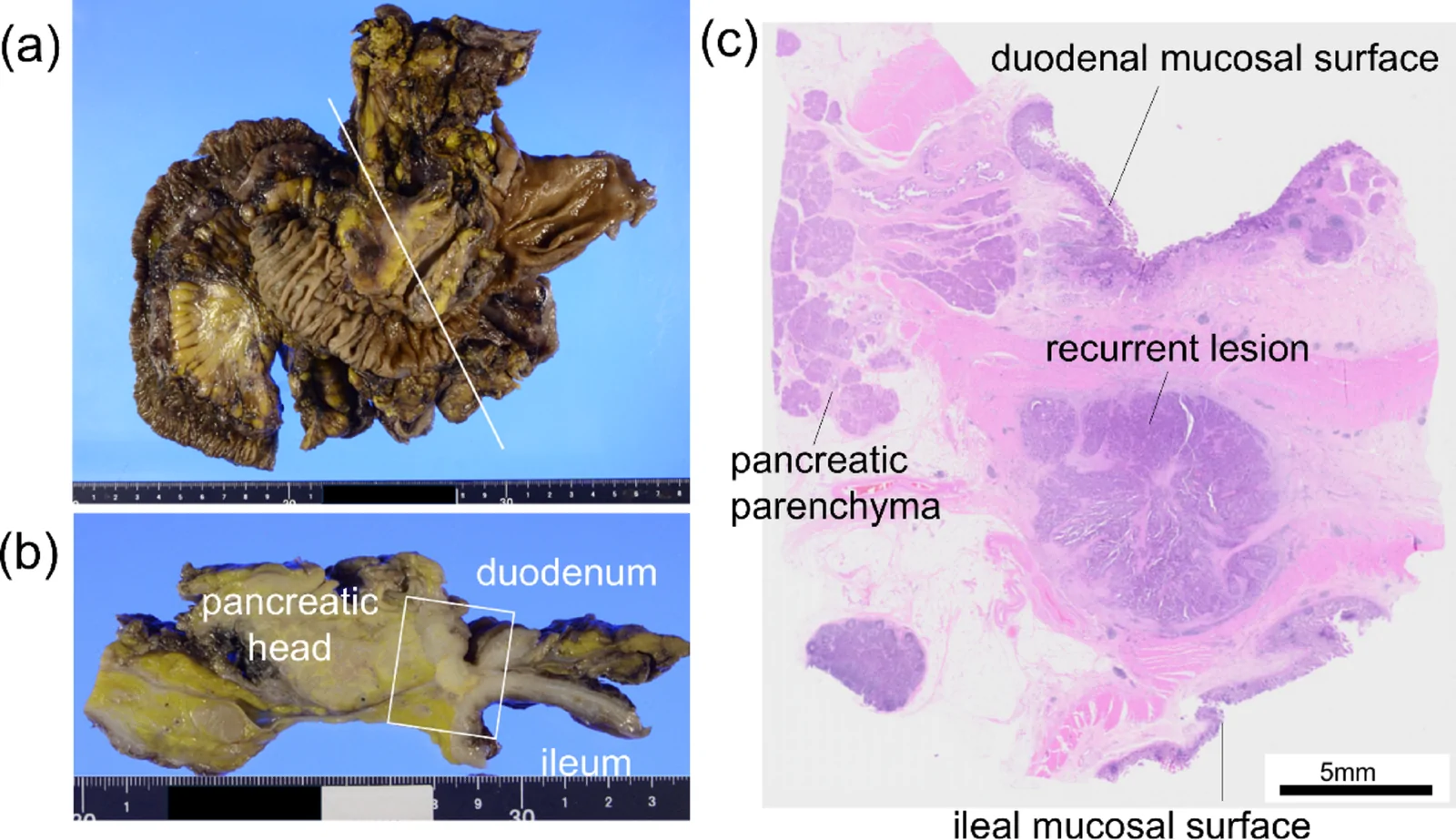
Macroscopic and loupe findings of the resected specimen. **a** Macroscopic view of the resected specimen. The white line indicates the cut line of the specimen corresponding to the images in (**b**) and (**c**). **b** The specimen was sectioned to evaluate the recurrent lesion located between the duodenum and the ileum. The white boxed area corresponds to the loupe image shown in (**c**). **c** A loupe-magnified histological image shows that the recurrent lesion extends to the muscularis propria of both the duodenum and the ileum. Although the pancreatic parenchyma is located close to the lesion, there is no clear evidence of direct invasion. The scale bar indicates 5 mm

**Fig. 7:**
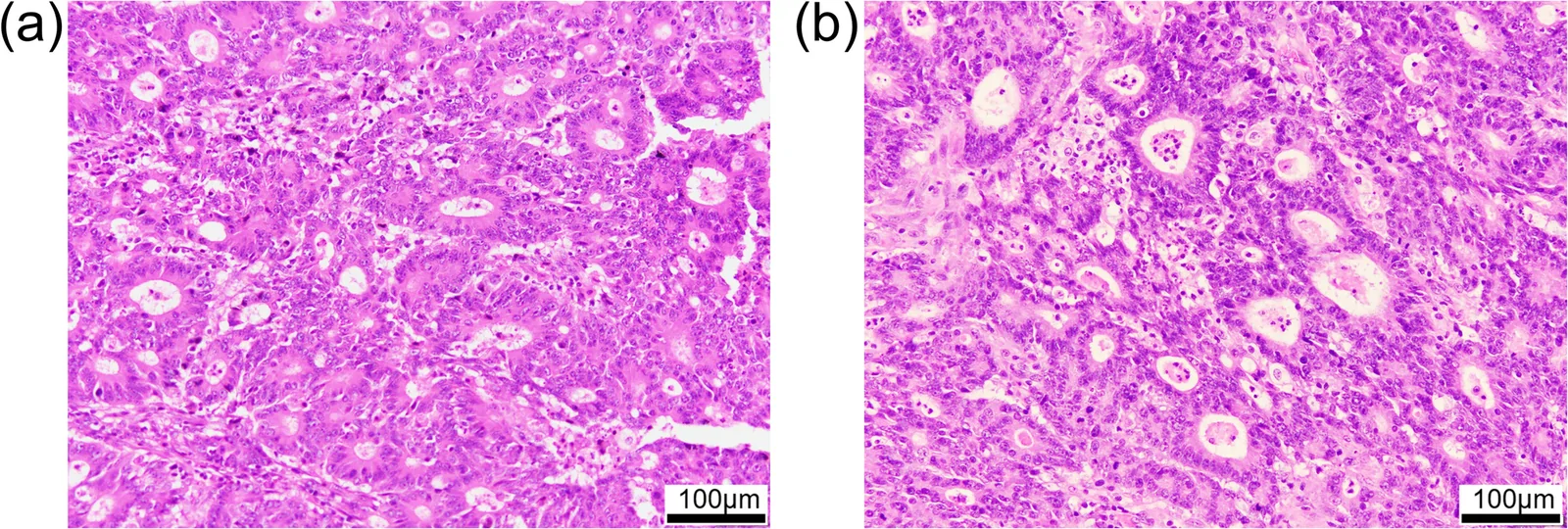
Histopathological comparison of the primary and recurrent tumors. **a** The hematoxylin and eosin-stained section of the primary tumor showed atypical cells with nuclear irregularity proliferating in fused glandular structures, consistent with moderately differentiated tubular adenocarcinoma. **b** The lesion resected by pancreaticoduodenectomy showed morphological features that were nearly identical to those of the primary tumor. Scale bars in both panels indicate 100 μm

**Fig. 8:**
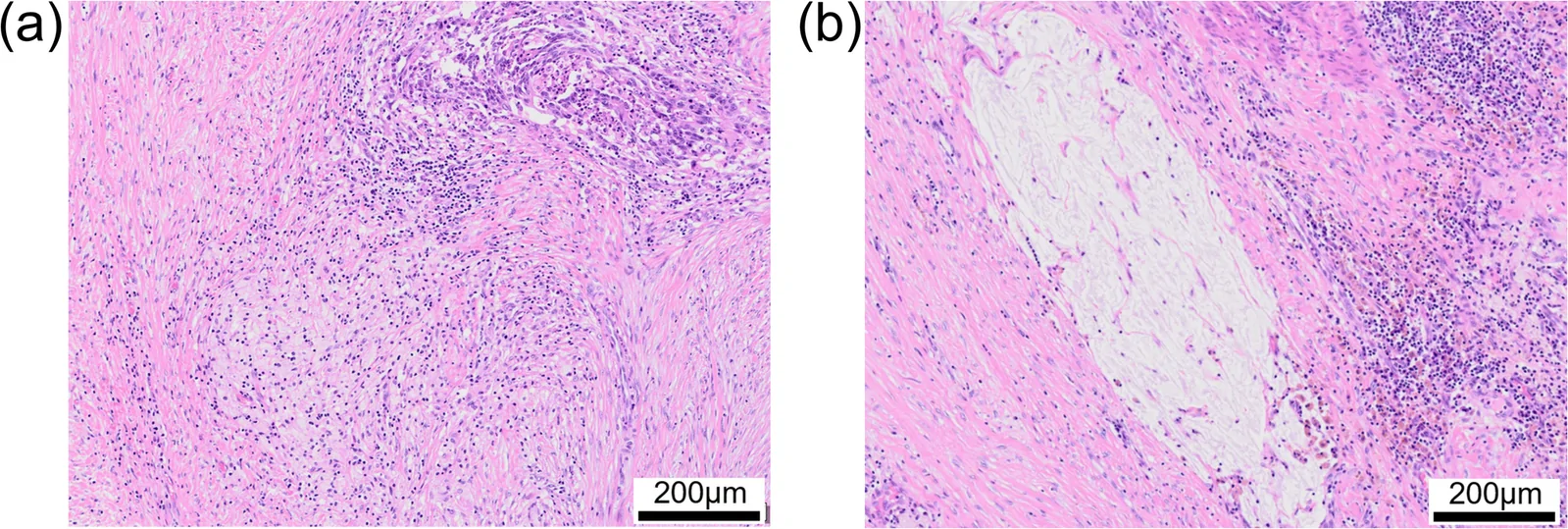
Histopathological findings indicating the effects of chemotherapy **a** In the peritumoral area, host tissue response to tumor cell disappearance was observed, characterized by xanthogranuloma-like features with infiltration of foamy histiocytes. **b** Areas of mucin lakes were also observed, which were considered to reflect tumor cell disappearance in response to chemotherapy. Scale bars in both panels indicate 200 μm

The operation time was 9 h and 50 min, with blood loss of 870 mL (Fig. 5). Postoperatively, bleeding from the gastrojejunostomy site was suspected; however, the patient improved with conservative management and was discharged 34 days after surgery. Histopathological examination of the resected specimen revealed that the adenocarcinoma lesions involved both the duodenum and ileum, infiltrating from the serosal side toward the muscularis propria. However, the mucosal layer was not involved, and no direct invasion of the pancreatic parenchyma was identified (Fig. 6a–c). No definitive lymph node structures were identified in the recurrent lesion. Comparison of the hematoxylin and eosin-stained sections of the primary tumor (Fig. 7a) and the specimen resected by pancreaticoduodenectomy (Fig. 7b) showed morphologically nearly identical differentiated tubular adenocarcinoma, consistent with recurrence. The dissection margins were negative. No lymph node metastasis or tumor involvement at the anastomosis was identified. In the peritumoral area, xanthogranuloma-like changes and the formation of mucin lakes were observed (Fig. 8a,b), which were considered to represent host tissue response associated with tumor cell disappearance after chemotherapy.

Postoperative adjuvant chemotherapy was not administered because the patient strongly expressed an aversion to the adverse effects. In this patient, serum CEA and CA19-9 levels remained within the normal ranges throughout the clinical course from the initial diagnosis. The patient has been followed up with regular CE-CT examinations, and more than 7 years have passed since the pancreaticoduodenectomy without any evidence of recurrence to date.

## Discussion

In general, recurrence after curative resection of the primary colorectal cancer is considered a manifestation of systemic disease. Systemic therapy is often recommended as the initial treatment. Surgical resection may be considered in cases where the recurrence is localized and disease control has been achieved; however, its therapeutic benefit remains unclear [2]. Complete resection of the recurrent lesion, when feasible, has been strongly associated with improved long-term survival. A previous study reported that patients who underwent curative resection achieved a 5-year survival rate of 58% and a median survival time of 66 months, whereas no patients in the incomplete resection group survived 5 years [7].

When pancreaticoduodenectomy is performed in association with colorectal cancer, the indications can be broadly classified into three clinical scenarios. Firstly, in cases of advanced ascending or transverse colon cancer that directly invades pancreatic parenchyma or the duodenum [3, 8, 9]. Secondly, in cases of metastasis to pancreatic parenchyma, several reports have described resection of metastatic pancreatic tumors from colorectal cancer [10–12]. Finally, pancreaticoduodenectomy can be performed to achieve resection of local recurrence as in our present case [3–5]. Retrospectively, this case may have been suitable for primary pancreaticoduodenectomy, as described in the first category. During initial surgery, numerous feeding vessels flowing from the duodenal side were identified, strongly suggesting duodenal invasion. However, the initial surgery was performed at a medium-sized community hospital, where an intraoperative shift to one-stage pancreaticoduodenectomy was considered high-risk and impractical, given the established association between institutional volume and postoperative outcomes [13]. Pancreaticoduodenectomy has become a procedure that can be performed safely in recent years; nevertheless, a perioperative mortality rate of approximately 2% has been documented [14]. This procedure should be undertaken in specialized centers that are fully equipped to manage postoperative pancreatic fistula and to provide emergent interventional radiologic treatment for pseudoaneurysmal hemorrhage [15], which may occur as a serious complication.

The mechanism and pattern of recurrence in the present case warrant careful consideration. Several mechanisms could explain the recurrent lesion, including regrowth of microscopic residual disease, peritoneal dissemination, intraoperative tumor cell spillage, and lymph node metastasis. Although pathological evaluation of the initial resection specimen indicated complete tumor removal, microscopic disease may have remained because, for example, the examined sections may not have included the true tumor front. With this possibility in mind, the clinicopathological findings in the present case were most consistent with regrowth of microscopic residual disease at the dissection plane of the initial operation for the following reasons.

First, the recurrent lesion was solitary and located precisely in the area where dissection had been technically challenging during the initial surgery because of suspected duodenal involvement. Second, histopathological examination demonstrated tumor infiltration into both the duodenum and ileum from the serosal side toward the muscularis propria without mucosal exposure, suggesting an extrinsic origin rather than a primary intraluminal process. Third, the lesion involved the adjacent duodenum and ileum to a similar extent, which would be difficult to explain by lymphatic spread alone. Finally, no residual lymph node architecture was identified within the recurrent lesion, and no lymph node metastases were detected elsewhere in the resected specimen. Although the involvement of other mechanisms cannot be excluded, taken together, these findings suggest that the recurrent tumor was likely arose from microscopic residual cancer cells at the dissection plane during the initial surgery. Complete resection was achieved by salvage pancreaticoduodenectomy, resulting in disease-free survival for more than 7 years and thereby supporting the validity of this treatment strategy.

When pancreaticoduodenectomy is performed in an atypical setting after major gastrointestinal resection, as in the present case, securing an adequate operative field is considered critically important. Because the ascending colon had already been mobilized from the retroperitoneum and resected with anastomosis during the initial surgery, the present procedure differed somewhat from the classic Cattell–Braasch maneuver [16]. However, by following the same principle and completely mobilizing the jejunum and ileum along their entire length, extensive direct visualization of the entire duodenum was achieved. The absence of pathological invasion into the pancreatic parenchyma and lymph node metastasis at the root of the ileal vessels suggests, in retrospect, that pancreaticoduodenectomy might have been avoided by performing partial duodenal resection and that a more limited extent of small-bowel resection might have been possible. However, no sufficient intraoperative evidence was available to justify omitting these extended resections. In addition, the residual small-bowel length was 170 cm, which is generally considered to carry a low risk of short bowel syndrome [17]. Therefore, given that achieving reliable oncological clearance was prioritized in this salvage setting, the operative procedure and extent of resection were considered acceptable. There have been reports of even more aggressive and risk-taking surgical approaches attempted for recurrent colorectal cancer.　Wo et al. reported an extracorporeal intestinal resection followed by pancreaticoduodenectomy and subsequent autotransplantation of the intestine [18]. Natsume et al. reported another challenging case in which hepatectomy was performed for a second recurrence after pancreaticoduodenectomy for recurrent colorectal cancer [4]. These procedures represent carefully planned attempts tailored to institutional expertise and resources.

We consider that complete resection was the principal contributor to the favorable long-term outcome in this case; however, the role of chemotherapy should also be addressed. Postoperative histopathological examination clearly demonstrated a sufficient therapeutic response to chemotherapy. These findings suggest that, after disease control has been achieved with chemotherapy for localized recurrence, salvage surgery involving a substantially more invasive procedure than the initial operation may be feasible in selected patients. In this case, Bevacizumab was included in the chemotherapy regimen. The possibility that bevacizumab contributed to postoperative gastrointestinal bleeding cannot be excluded. Nevertheless, close interinstitutional coordination enabled discontinuation of bevacizumab as soon as surgery was considered, thereby ensuring the recommended six-week washout period [19]. Therefore, the preoperative management was considered appropriate. Regarding postoperative adjuvant chemotherapy following complete resection, Noda et al. reported that among 49 patients who underwent resection with complete tumor-free margins for peritoneal recurrence of colorectal cancer, those who received adjuvant chemotherapy had significantly better survival outcomes compared with those who did not [20]. These findings suggest a potential therapeutic benefit of postoperative adjuvant chemotherapy. However, because adjuvant chemotherapy was not administered at the patient’s request, its significance cannot be evaluated in this case.

In conclusion, this case is noteworthy because long-term disease-free survival was achieved through aggressive surgical resection combined with preoperative chemotherapy for localized recurrence of ascending colon cancer. This case also underscores the clinical value of close collaboration between a regional community hospital and a tertiary referral center.

## Data Availability

Imaging findings and pathological data related to this case are available from the corresponding author upon reasonable request.

## Abbreviations

^18^F-FDG-PET: ^18^F-fluorodeoxyglucose positron emission tomography
CE-CT: Contrast-enhanced computed tomography
FOLFIRI + bev: 5-Fluorouracil, irinotecan, leucovorin, and bevacizumab
XELOX: Oxaliplatin and capecitabine
